# Supplementary material for: Molecular and clinical analyses of PHF6 mutant myeloid neoplasia provide their pathogenesis and therapeutic targeting
Source: Nat Commun. 2024 Feb 28;15:1832. doi: 10.1038/s41467-024-46134-w (PMC10901781; doi:10.1038/s41467-024-46134-w)
Supplement: Supplementary file 1 — Supplementary Information [file 41467_2024_46134_MOESM1_ESM.pdf]

Supplementary Information

Molecular and clinical analyses of PHF6 mutant myeloid neoplasia provide their pathogenesis and therapeutic targeting

Yasuo Kubota[1], Xiaorong Gu[1], Laila Terkawi[1], Juraj Bodo[2], Bartlomiej P. Przychodzen[1], Hussein Awada[1], Nakisha Williams[1], Carmelo Gurnari[1,3], Naomi Kawashima[1], Mai Aly[1], Arda Durmaz[1], Minako Mori[1], Ben Ponvilawan[1], Tariq Kewan[1], Waled Bahaj[1], Manja Meggendorfer[4], Babal K. Jha[1,5], Valeria Visconte[1], Heesun J Rogers[2], Torsten Haferlach[4], Jaroslaw P. Maciejewski[1]

1. Department of Translational Hematology and Oncology Research, Taussig Cancer Institute, Cleveland Clinic, Cleveland, OH, USA.
2. Department of Laboratory Medicine, Cleveland Clinic, Cleveland Clinic, Cleveland, OH, USA.
3. Department of Biomedicine and Prevention, University of Rome Tor Vergata, Rome, Italy.
4. MLL Munich Leukemia Laboratory, Munich, Germany.
5. Center for Immunotherapy and Precision Immuno-Oncology, Lerner Research Institute (LRI) Cleveland Clinic, Cleveland, OH, USA.

Inventory of Supplementary Information

Supplementary Figure 1: Lollipop plot illustrating PHF6 mutational data in this study group.
Supplementary Figure 2: The frequencies of PHF6 mutations in MNs and their subtypes.
Supplementary Figure 3: PHF6 status in femal AML cases.
Supplementary Figure 4: Comparisons of frequencies of mutations in nonescpaing genes based on X chromosomal status.
Supplementary Figure 5: Comparisons of frequencies of mutations with or without RUNX1 mutations based on each MN disease.
Supplementary Figure 6: Significant genetics correlations by each sex.
Supplementary Figure 7: Survival analysis for AML cases with or without PHF6 mutations by each cohort.
Supplementary Figure 8: Event free survival for AML cases with or withous PHF6 mutations in the CCF and MLL cohort.
Supplementary Figure 9: Survival analysis for AML cases by disease risk.
Supplementary Figure 10: Survival analysis for AML cases with double mutations (PHF6 and RUNX1) by each cohort.
Supplementary Figure 11: Event free survival for AML cases with double mutations (PHF6 and RUNX1) in the CCF and MLL cohort.
Supplementary Figure 12: SDS-PAGE for IP-WB.
Supplementary FIgure 13: Gene expression analysis in RUNX1-mutated AML cases.
Supplementary Figure 14: The fraction of truncating mutations in PHF6- and/or RUNX1-mutated AML cases.
Supplementary FIgure 15: Western blots of PHF6 IP.
Supplementary Figure 16: Western blots of endogenous PHF6 IP.
Supplementary Figure 17: Lollipop plot showing RUNX1 stopgain mutations in male AML cases.
Supplementary Figure 18: Comparisons between PHF6- and/or RUNX1-mutated AML cases.
Supplementary Table 1: Test statistics for mutations in genes on X chromosome.
Supplementary Table 2: Upregulated pathways in PHF6-mutated AML.
Supplementary Table 3: Downregulated pathways in RUNX1-mutated AML.
Supplementary Table 4: FCM results in patients' with PHF6 mutations.
Supplementary Table 5: The number of cases by each cohort.
Supplementary Table 6: Targeted panel genes.

Supplementary Figure 1

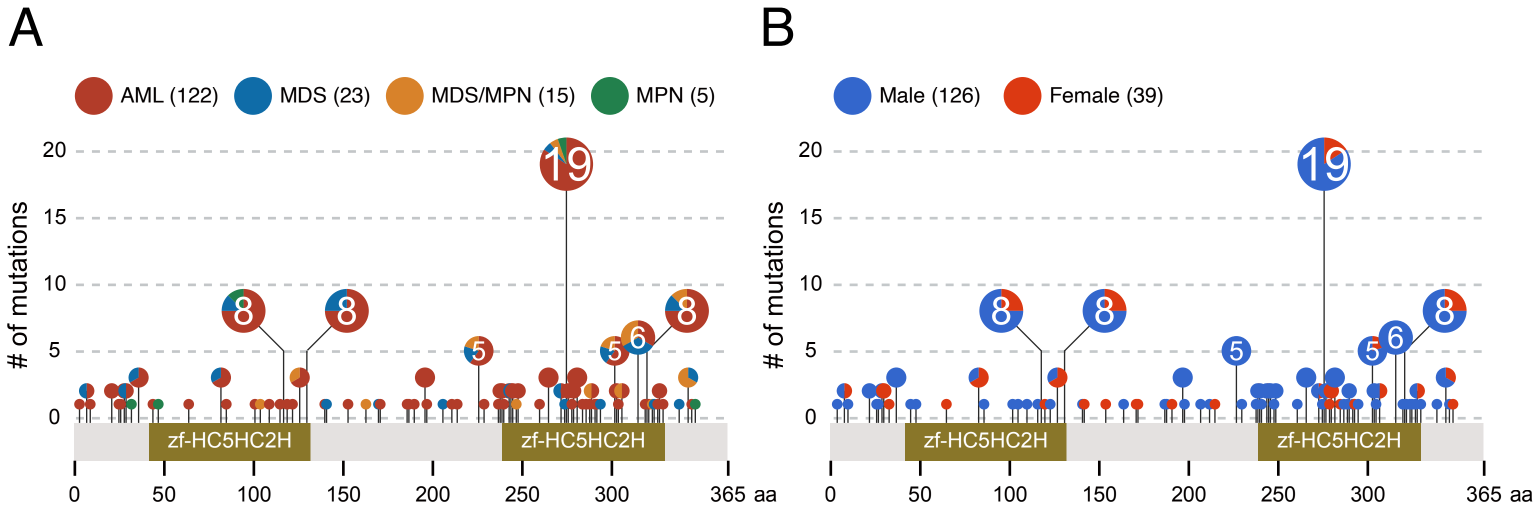

Supplementary Figure 1

Lollipop plot illustrating *PHF6* mutational data in this study group.

(A) Disease types and (B) sexes are shown by colors as indicated. The numbers in circles indicate the number of cases.

Supplementary Figure 2

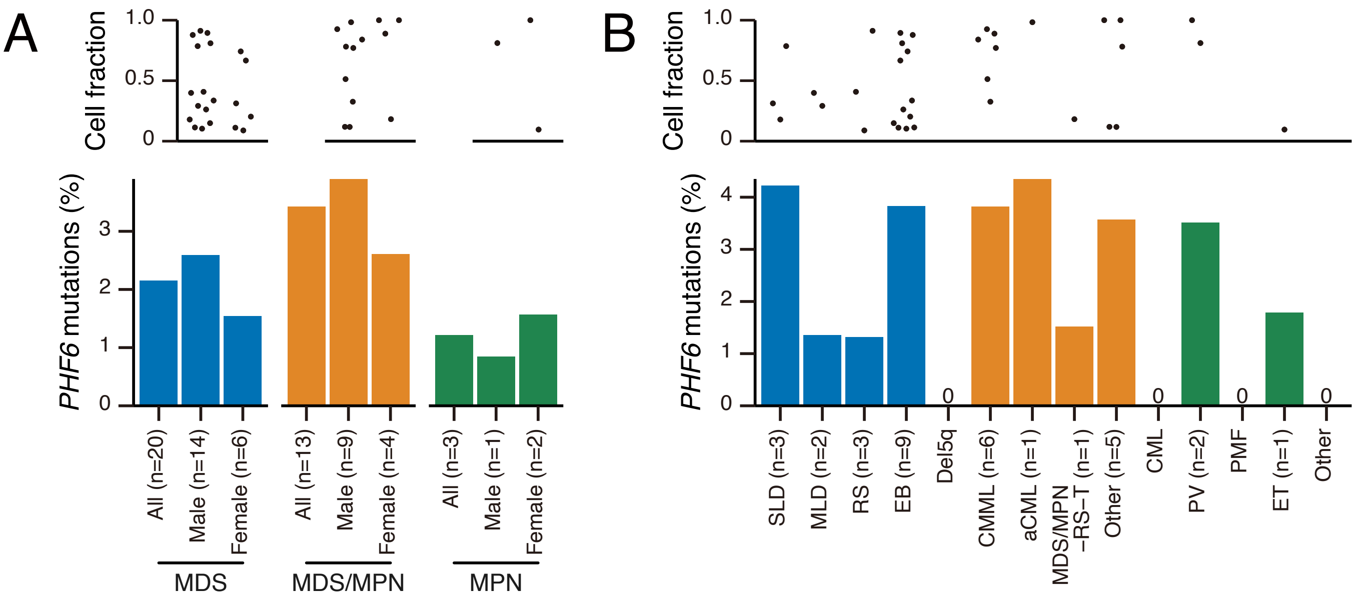

Supplementary Figure 2

(A) Comparisons of frequencies of *PHF6* mutations based on MN disease in each sex. Each dot in the upper panel represents the cell fraction with *PHF6* mutation.

(B) Comparisons of frequencies of *PHF6* mutations based on each MN subtype. Each dot in the upper panel represents the cell fraction with *PHF6* mutation.

Supplementary Figure 3

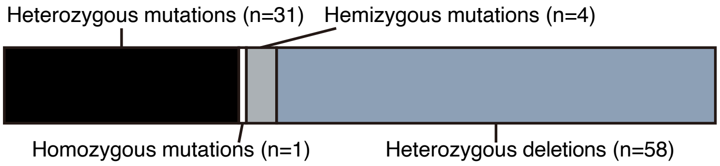

Supplementary Figure 3

PHF6 status in female AML cases.

Supplementary Figure 4

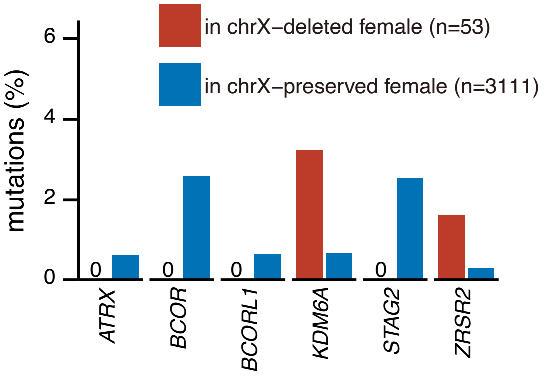

Supplementary Figure 4

Comparisons of frequencies of mutations in nonescaping genes based on X chromosomal status.

Supplementary Figure 5

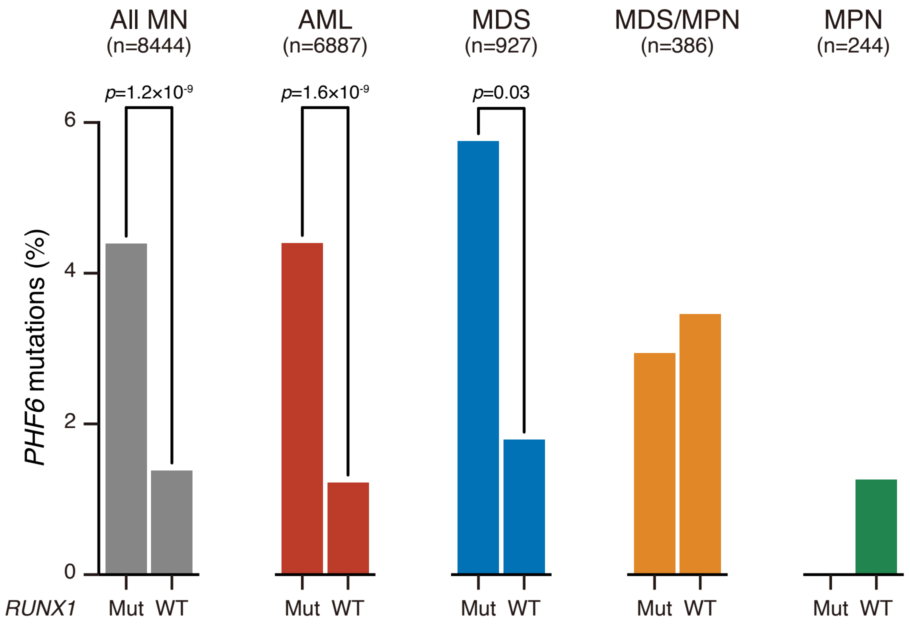

Supplementary Figure 5

Comparisons of frequencies of *PHF6* mutations with or without *RUNX1* mutations based on each MN disease. *p* values are from the two-sided Fisher's exact test and considered statistically significant at $p < 0.05$.

Supplementary Figure 6

A

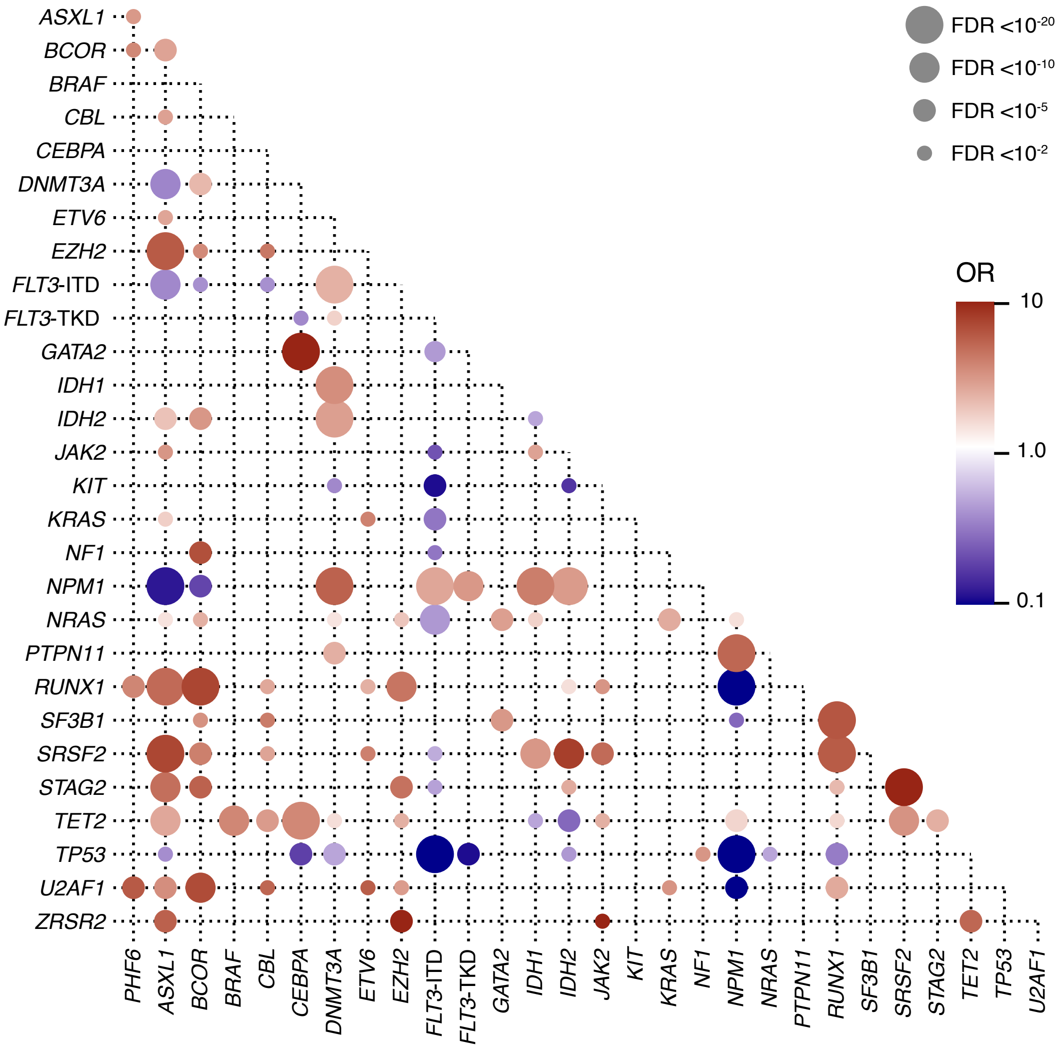

B

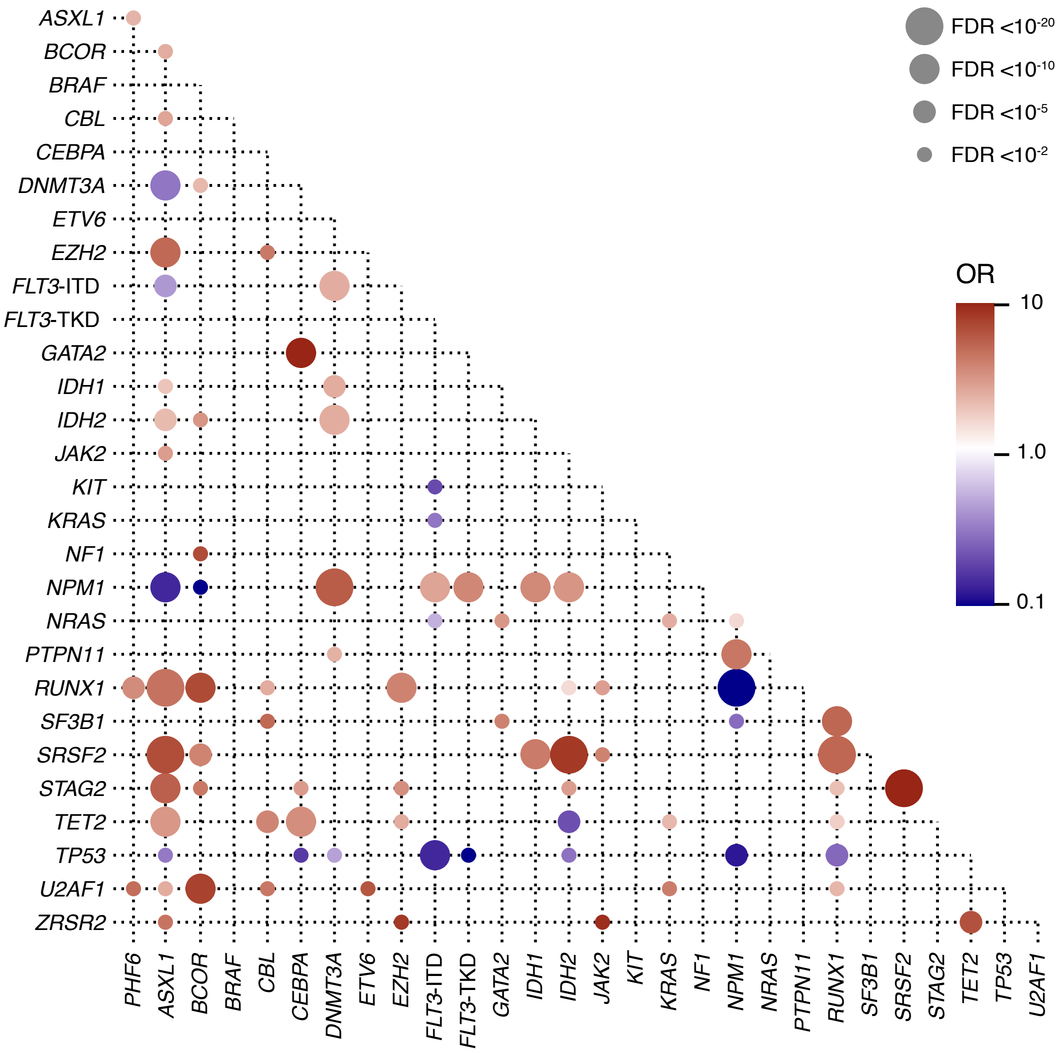

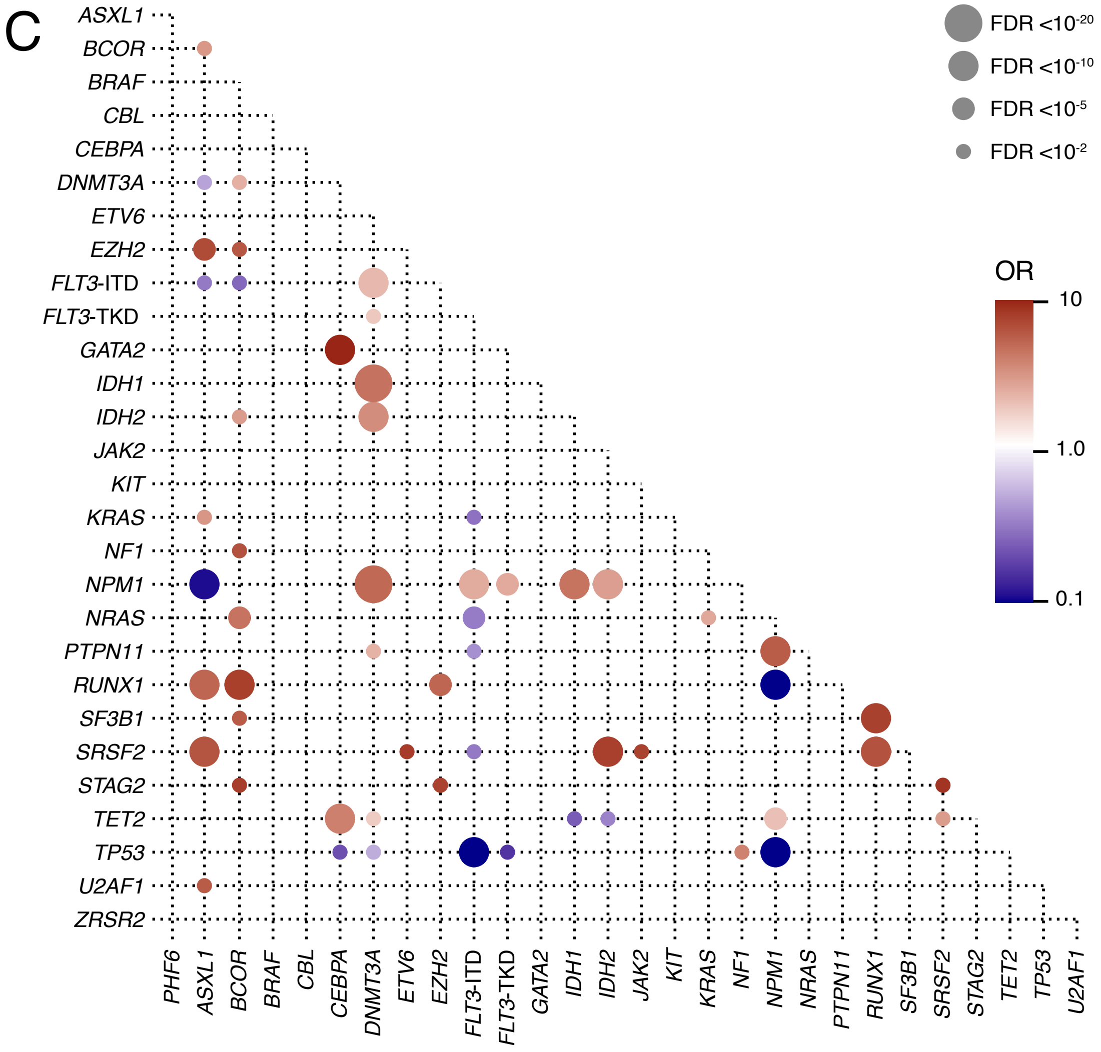

Supplementary Figure 6 Supplementary Figure

Bubble plot showcasing statistically significant (FDR < 0.05) positive (red) and negative (blue) correlations across gene group in all (A; n=6887), male (B; n=3715), and female (C; n=3172) cases.

Correlations across gene groups were assessed by Fisher's exact test and corrected by employing the Benjamini-Hochberg method.

Supplementary Figure 7

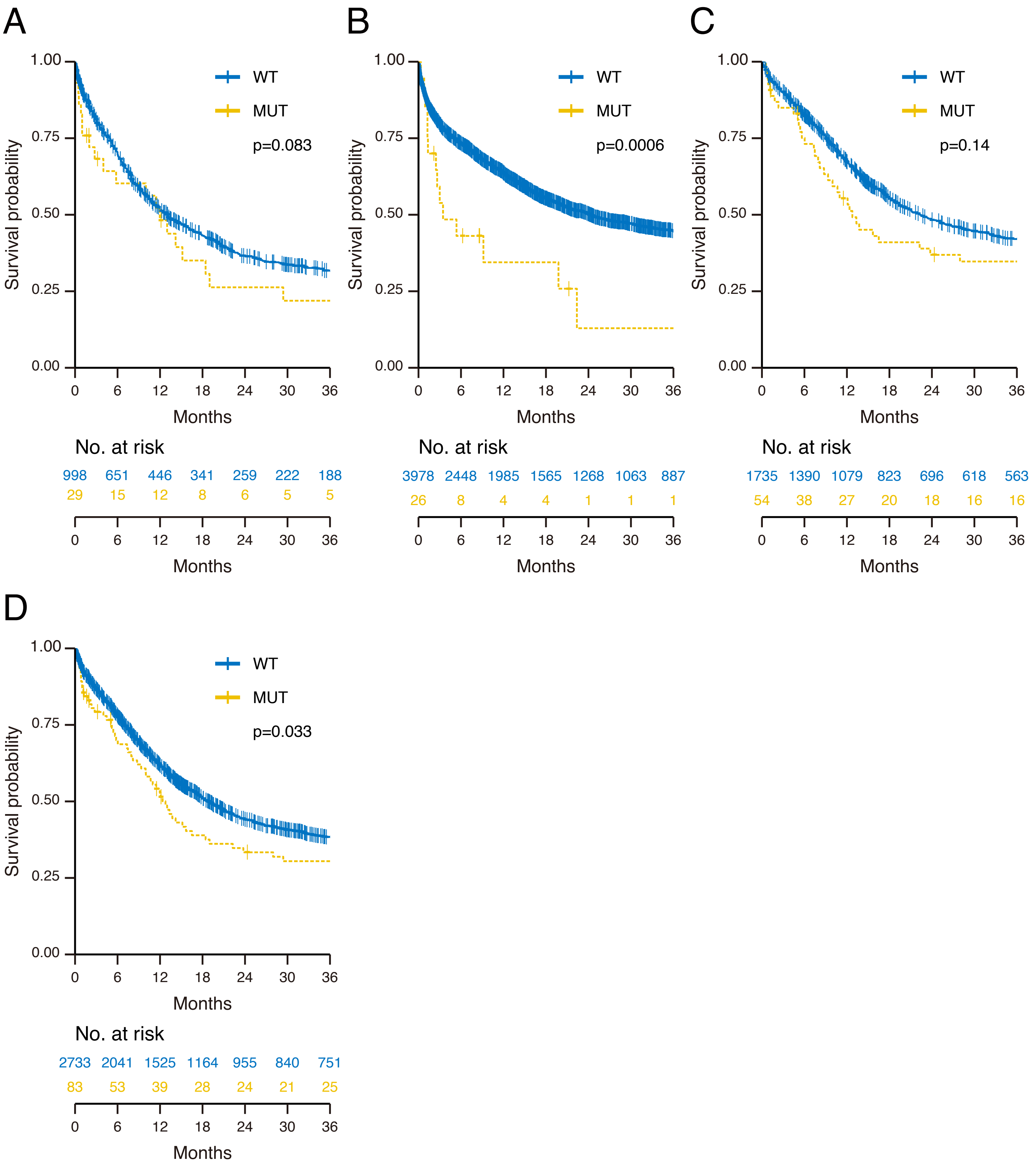

Supplementary Figure 7

Survival analysis for AML cases with or without *PHF6* mutations by each cohort.

Kaplan-Meier survival curves of overall survival for AML in CCF (A), MLL (B), open data (C), and CCF plus open data (D) cohort. For survival analysis, survival was estimated using the Kaplan-Meier method and the log-rank test was used to assess differences between groups.

Supplementary Figure 8

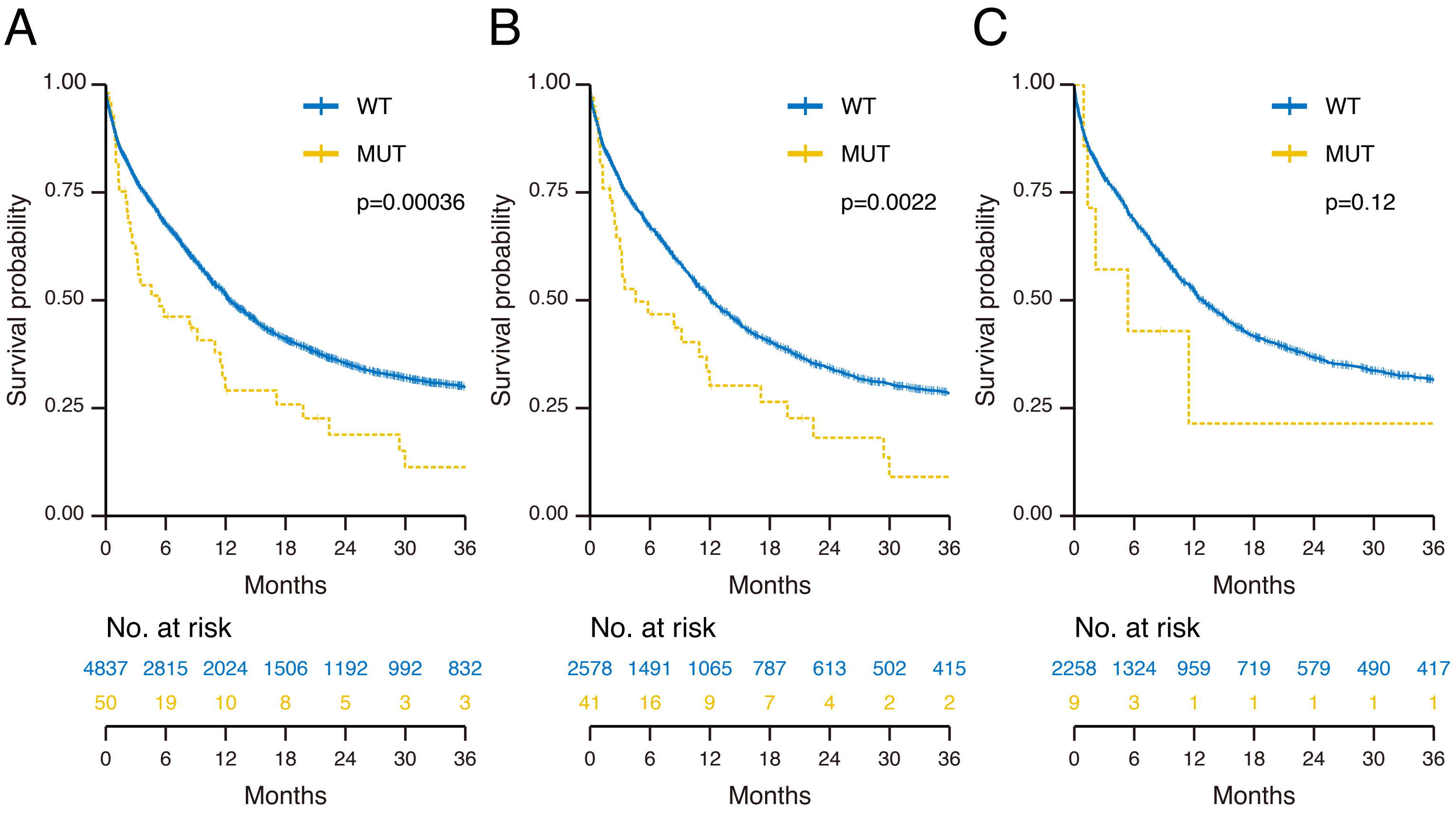

Supplementary Figure 8

Event free survival for AML cases with or without *PHF6* mutations in the CCF and MLL cohort. Kaplan-Meier survival curves of event free survival for AML in all (A), male (B), and female (C) cases. For survival analysis, survival was estimated using the Kaplan-Meier method and the log-rank test was used to assess differences between groups.

Supplematary Figure 9

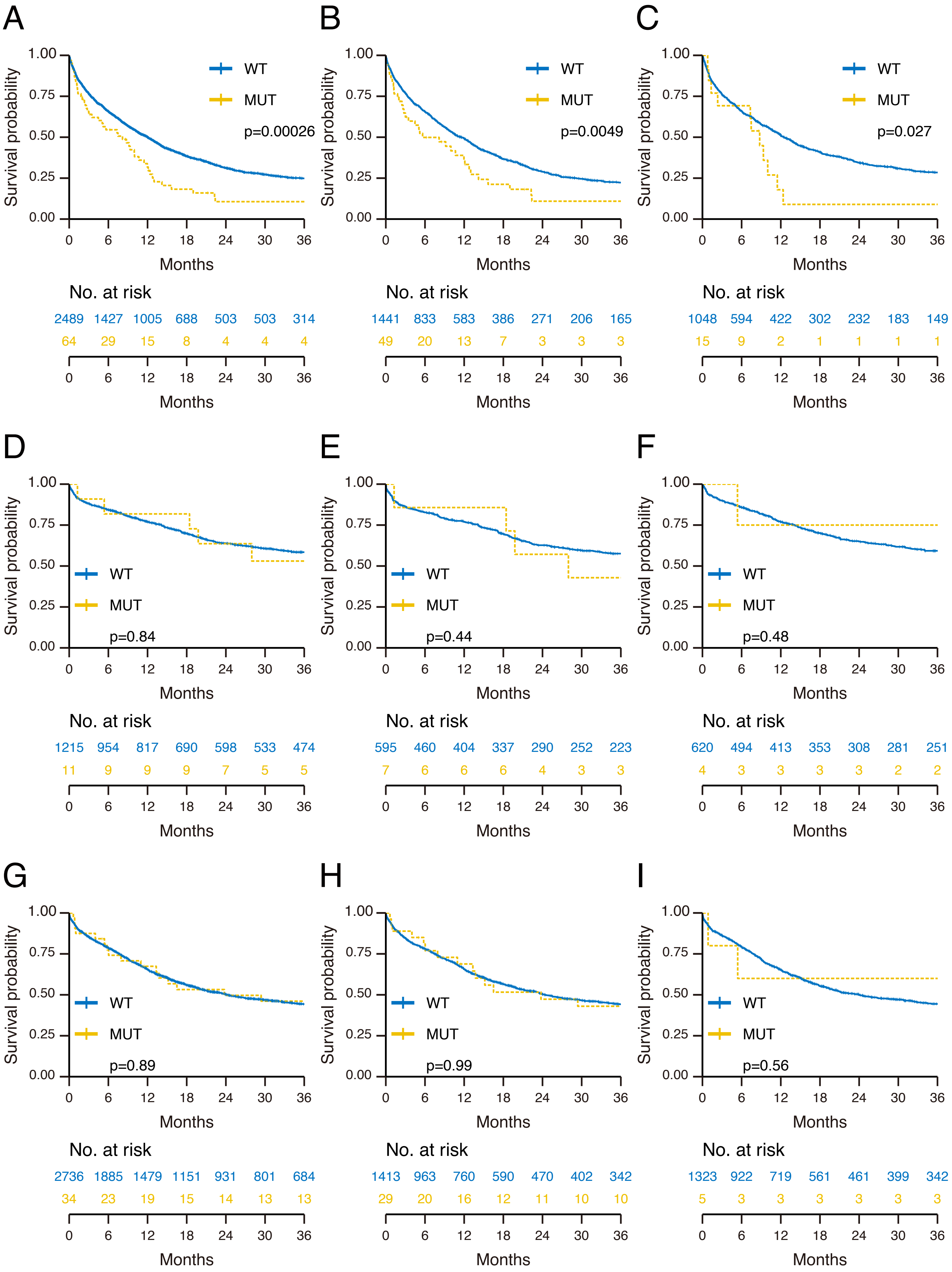

Supplementary Figure 9

Survival analysis for AML cases by disease risk.

Kaplan-Meier survival curves of overall survival for adverse risk AML in all cases (A), male cases (B), female cases (C), favorable risk in all cases (D), male cases (E), female cases (F), intermediate risk in all cases (G), male cases (H), and female cases(I). For survival analysis, survival was estimated using the Kaplan-Meier method and the log-rank test was used to assess differences between groups.

Supplementary Figure 10

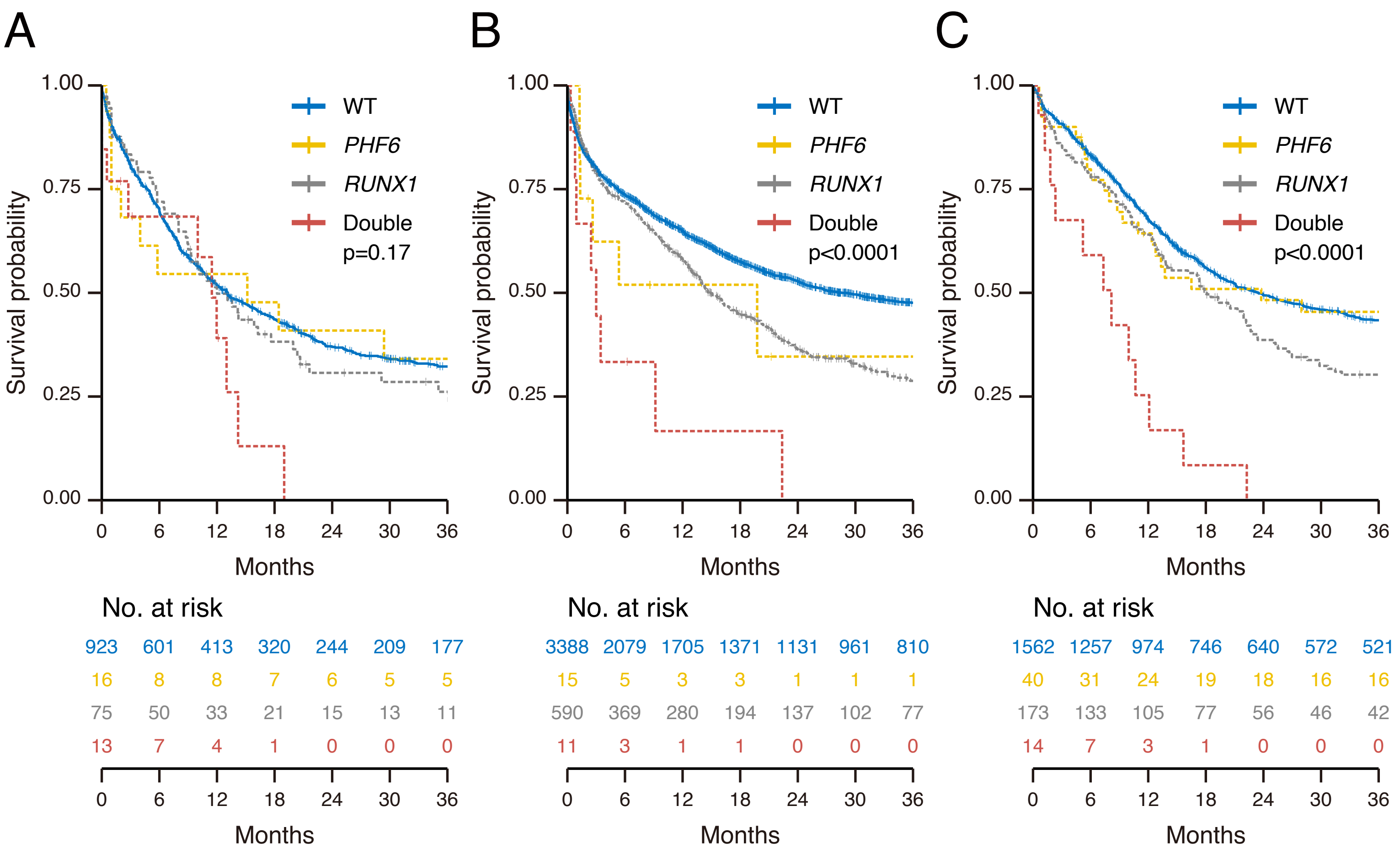

Supplementary Figure 10

Survival analysis for AML cases with double mutations (*PHF6* and *RUNX1*) by each cohort.

Kaplan-Meier survival curves of overall survival for AML in CCF (A), MLL (B), and open data (C) cohort.

For survival analysis, survival was estimated using the Kaplan-Meier method and the log-rank test was used to assess differences between groups.

Supplementary Figure 11

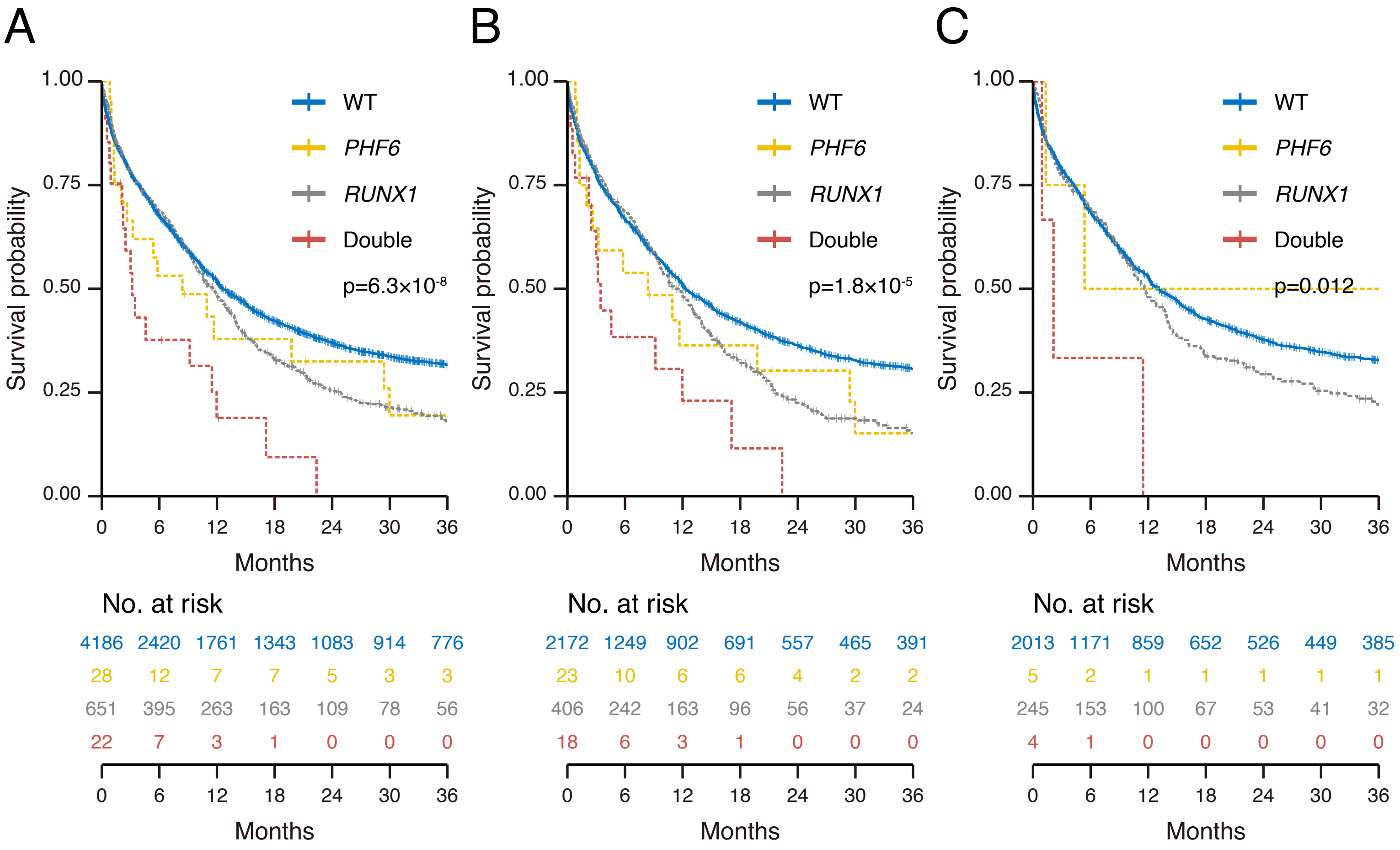

Supplementary Figure 11

Event free survival for AML cases with double mutations (*PHF6* and *RUNX1*) in the CCF and MLL cohort.

Kaplan-Meier survival curves of event free survival for female AML cases with double mutations (*PHF6* and *RUNX1*), single mutations, and negative cases in all (A), male (B), and female (C) cases.

For survival analysis, survival was estimated using the Kaplan-Meier method and the log-rank test was used to assess differences between groups.

Supplementary Figure 12

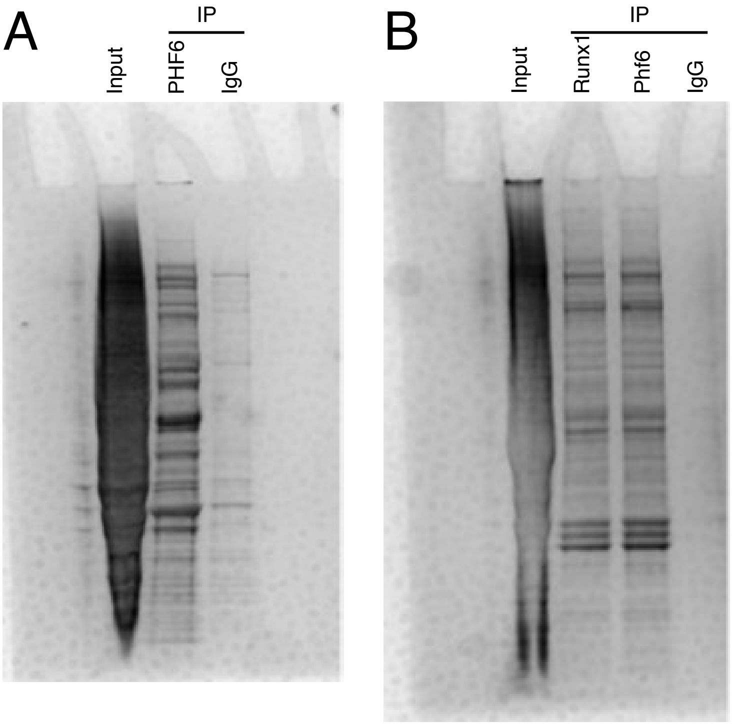

Supplementary Figure 12

(A) 1D SDS-PAGE for IP-WB in Figure 6C. (B)1D SDS-PAGE for IP-WB in Figure 6D. Proteins were visualized with Coomassie Blue staining. PHF6 / Phf6 and Runx1 IP clearly showed enrichments of selected proteins from the input.

Supplementary Figure 13

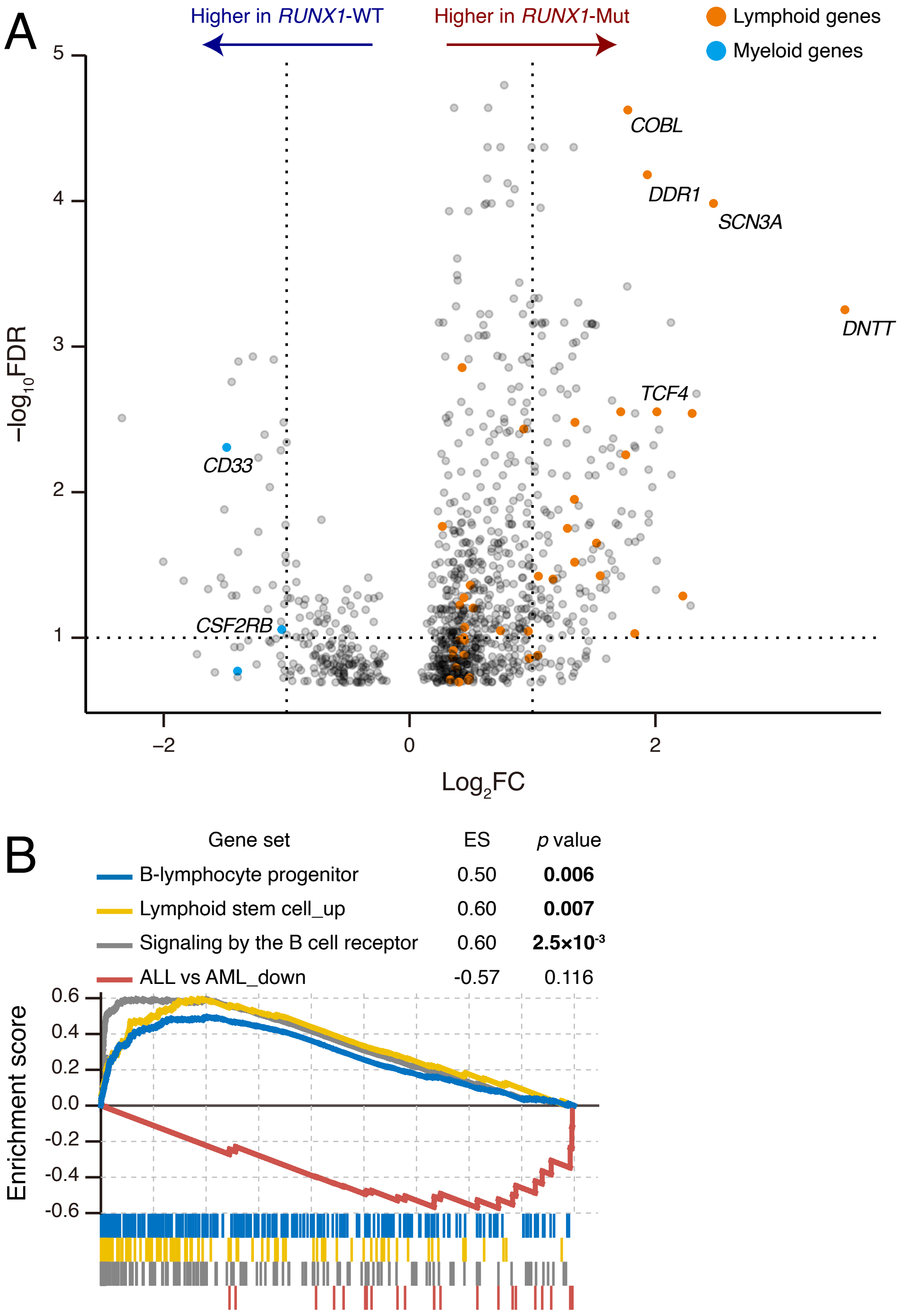

Supplementary Figure 13

(A) Volcano plot comparing significant expression difference between *RUNX1*-mutated (n=17) and wild-type (n=115) AML. Lymphoid and myeloid genes with differentially expression are colored in orange and blue, respectively. For differential expression gene analysis, we used the Bayesian method by the linear models for microarray expression data (limma) package version 3.50.0 in R software.

(B) GSEA plot showing changes in lymphoid and myeloid signature genes between *RUNX1*-mutated and wild-type AML. To perform the enrichment of difference between *RUNX1*-mutated and wild-type AML, we used the Gene Set Enrichment (GSEA) software (v.4.3.2).

Supplementary Figure 14

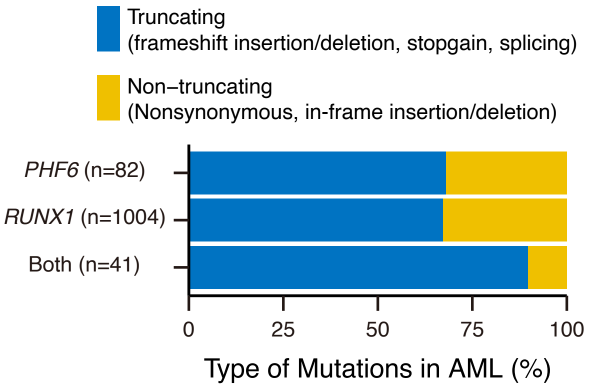

Supplementary Figure 14

The fraction of truncating and non-truncating mutations in *PHF6*-mutated, *RUNX1*-mutated, and both-mutated samples. Samples with mutations in both genes have at least one truncating mutation in either *PHF6* or *RUNX1*.

Supplementary Figure 15

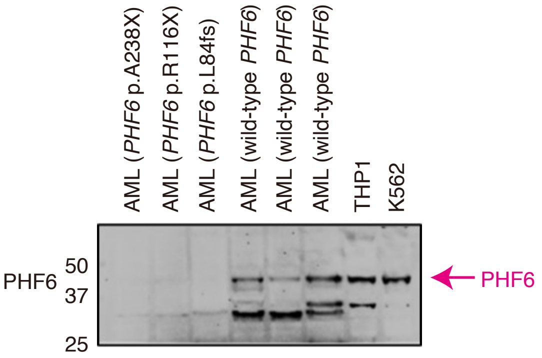

Supplementary Figure 15

Western blots of PHF6 IP of nuclear protein extracts in AML samples with PHF6 mutations or wild-types. PHF6 bands are indicated by arrow in red color. The Western blot was done once in THP-1 and K562 cells.

Supplementary Figure 16

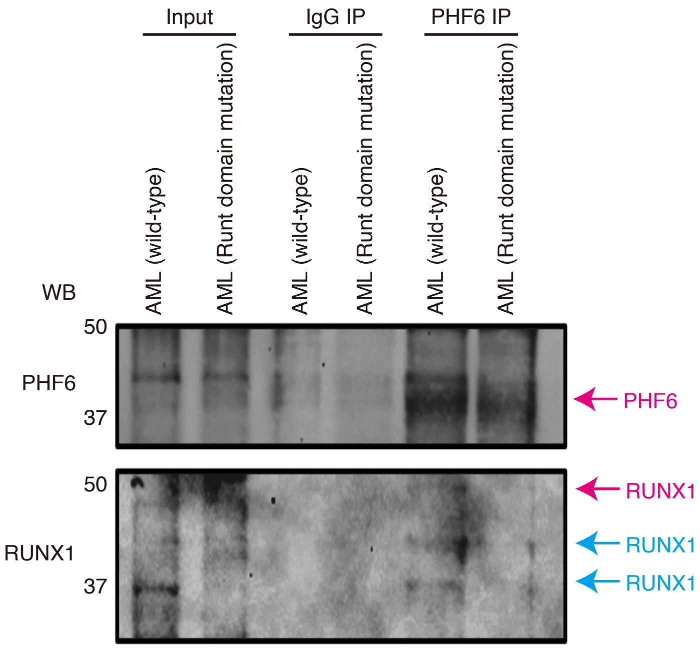

Supplementary Figure 16

Western blots of endogenous PHF6 IP of nuclear protein extracts in AML samples with wild-type *RUNX1* or Runt domain mutations. 5% input, IgG control IP, and PHF6 IP product were run side by side. The same membrane was probed with an anti-rabbit monoclonal antibody to PHF6 and an antimouse monoclonal antibody to RUNX1. PHF6 and RUNX1 bands are indicated by arrows in red color. The other RUNX1 isoforms bands are indicated by arrows in blue color. The Western blot was done once in these AML cells.

Supplementary Figure 17

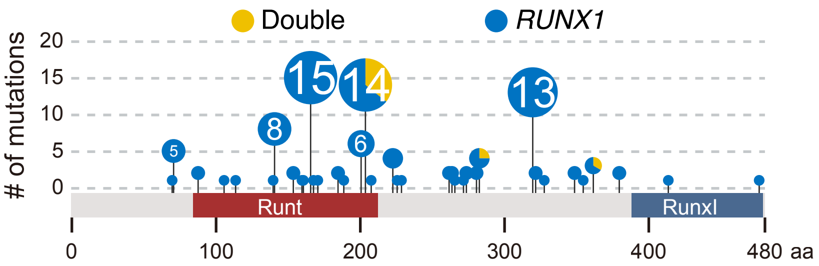

Supplementary Figure 17

Lollipop plot showing *RUNX1* stopgain mutations in male AML samples with double (*RUNX1* and *PHF6*) mutations, or only *RUNX1* mutations. Mutational groups are shown by colors as indicated. The numbers in circles indicate the number of cases.

Supplementary Figure 18

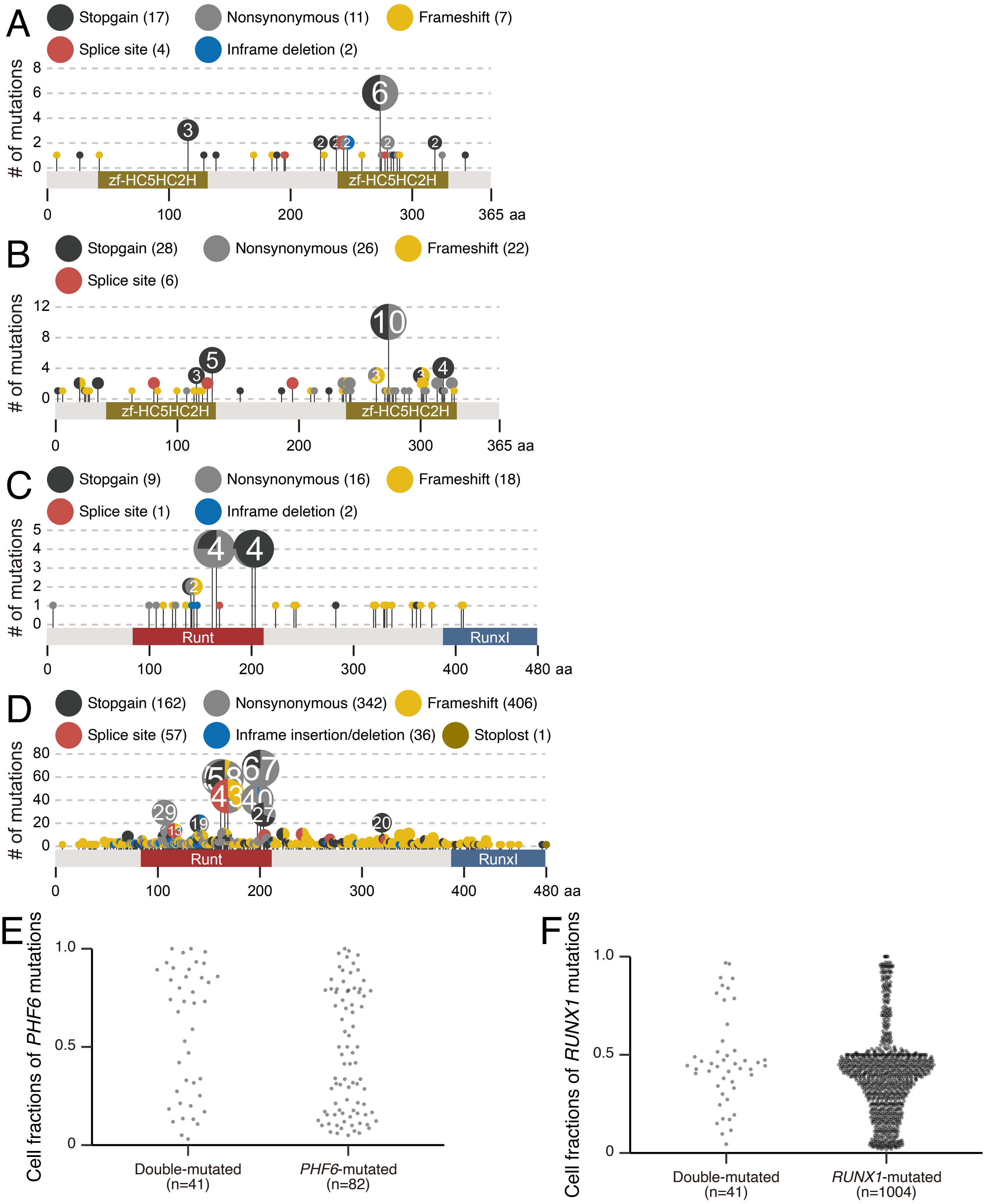

Supplementary Figure 18

Lollipop plot of *PHF6* mutations in case with *PHF6* and *RUNX1* mutations (A) and only *PHF6* mutations (B). Lollipop plot of *RUNX1* mutations in cases with *PHF6* and *RUNX1* mutations (C) and only *RUNX1* mutations (D). The cell fraction with mutated *PHF6* or *RUNX1* allele in cases with *PHF6* and *RUNX1* mutations (E) and *PHF6* or *RUNX1* mutations (F).

Supplementary Table 1: Test statistics for mutations in genes on X chromosome

Gene	Odds ratio	95% CI	*p* value	FDR
PHF6	3.02	1.93 - 4.72	2.43×10^{-7}	2.19×10^{-6}
*UBA1**	14.0	1.20 - 163	3.29×10^{-2}	7.40×10^{-2}
ZRSR2	4.31	2.18 - 8.52	2.70×10^{-6}	1.22×10^{-5}
STAG2	1.79	1.32 - 2.43	1.57×10^{-4}	4.71×10^{-4}
BCORL1	1.76	0.537 - 5.76	0.409	0.525
BCOR	1.29	0.957 - 1.74	0.100	0.180
ATRX	1.13	0.252 - 5.07	1	1
PIGA	1.04	0.834 - 1.30	0.780	0.877
KDM6A	0.592	0.292 - 1.20	0.157	0.236

*To avoid dividing zero, pseudo count of 1 is added to each cell in 2x2 table.

Supplementary Table 2: Upregulated pathways in *PHF6*-mutated AML

Pathway	Size		NES	NOM p-val	FDR q-val	FWER p-val	Rank at max	Leading edge
VALK_AML_CLUSTER_1	29	0.67682016	2.0028014	0	1	0.495	2216	tags=55%, list=12%, signal=63%
BOGNI_TREATMENT_RELATED_MYELOID_LEUKEMIA_DN	33	0.5629568	1.8665005	0.012048192	1	0.815	5363	tags=64%, list=30%, signal=90%
REACTOME_RUNX2_REGULATES_BONE_DEVELOPMENT	29	0.5176277	1.8553703	0.0072202166	1	0.834	1623	tags=41%, list=9%, signal=45%
HADDAD_B_LYMPHOCYTE_PROGENITOR	274	0.46060374	1.8315852	0.014836796	1	0.866	4305	tags=45%, list=24%, signal=58%
CORONEL_RFX7_DIRECT_TARGETS_UP	43	0.4852861	1.8039889	0.0027027028	1	0.902	2722	tags=40%, list=15%, signal=46%
WP_HEMATOPOIETIC_STEM_CELL_GENE_REGULATION_BY_GABP_ALPHABETA_COMPLEX	21	0.60628396	1.7770096	0.014150944	1	0.923	3229	tags=52%, list=18%, signal=64%
FARMER_BREAST_CANCER_CLUSTER_5	19	0.6610508	1.7377043	0.016032064	1	0.958	3559	tags=47%, list=20%, signal=59%
KEGG_BASAL_TRANSCRIPTION_FACTORS	32	0.53525555	1.7120938	0.020703934	1	0.97	2322	tags=38%, list=13%, signal=43%
DAZARD_UV_RESPONSE_CLUSTER_G6	142	0.4376627	1.7111005	0.027842227	1	0.97	4775	tags=49%, list=27%, signal=66%
DAZARD_RESPONSE_TO_UV_NHEK_DN	294	0.41728464	1.655613	0.03211009	1	0.988	4695	tags=48%, list=26%, signal=64%
BIOCARTA_RAC1_PATHWAY	20	0.496195	1.6541853	0.021686748	1	0.99	1795	tags=35%, list=10%, signal=39%
YAO_TEMPORAL_RESPONSE_TO_PROGESTERONE_CLUSTER_2	45	0.42334962	1.652738	0.016216217	1	0.99	2908	tags=38%, list=16%, signal=45%
MEISSNER_NPC_ICP_WITH_H3K4ME3	19	0.5282084	1.6264662	0.016528925	1	0.991	3779	tags=53%, list=21%, signal=67%
REACTOME_RUNX2_REGULATES_OSTEOBLAST_DIFFERENTIATION	23	0.47860286	1.6218047	0.026936026	1	0.993	1623	tags=39%, list=9%, signal=43%
YUAN_ZNF143_PARTNERS	21	0.58038586	1.6008521	0.04863813	1	0.995	5322	tags=52%, list=30%, signal=74%
STAMBOLSKY_TARGETS_OF_MUTATED_TP53_UP	42	0.39657044	1.5962424	0.012594459	1	0.995	3159	tags=38%, list=18%, signal=46%
REACTOME_CONSTITUTIVE_SIGNALING_BY_EGFRVIII	15	0.5440623	1.585723	0.045212764	1	0.995	1143	tags=33%, list=6%, signal=36%
LINDGREN_BLADDER_CANCER_CLUSTER_1_UP	116	0.3728909	1.5791982	0.014251782	1	0.996	4023	tags=40%, list=22%, signal=51%
BIOCARTA_CTCF_PATHWAY	24	0.50041837	1.5755477	0.046753246	1	0.997	4076	tags=58%, list=23%, signal=75%
BYSTROEM_CORRELATED_WITH_IL5_DN	60	0.44670147	1.5701308	0.033783782	1	0.997	3699	tags=45%, list=21%, signal=56%
OSMAN_BLADDER_CANCER_DN	420	0.37290838	1.5638547	0.03908046	1	0.997	5067	tags=46%, list=28%, signal=62%
FIGUEROA_AML_METHYLATION_CLUSTER_2_UP	46	0.40709114	1.5517147	0.031073445	1	0.997	3579	tags=39%, list=20%, signal=49%
PID_SMAD2_3NUCLEAR_PATHWAY	76	0.37118644	1.5437632	0.025568182	1	0.997	5020	tags=46%, list=28%, signal=64%
PID_IL2_PI3K_PATHWAY	33	0.4148004	1.532098	0.044619422	1	0.998	4076	tags=42%, list=23%, signal=55%
GENTILE_UV_LOW_DOSE_UP	25	0.46566504	1.5169954	0.048231512	1	0.999	4218	tags=44%, list=23%, signal=57%
GAL_LEUKEMIC_STEM_CELL_UP	117	0.34872118	1.5157202	0.015337423	1	0.999	3617	tags=33%, list=20%, signal=41%
PID_AR_TF_PATHWAY	46	0.40596524	1.5073072	0.045783132	1	0.999	2561	tags=33%, list=14%, signal=38%
BILD_CTNNB1_ONCOGENIC_SIGNATURE	74	0.3902668	1.4852865	0.031400967	1	0.999	3438	tags=35%, list=19%, signal=43%
REACTOME_NR1H3_NR1H2_REGULATE_GENE_EXPRESSION_LINKED_TO_CHOLESTEROL_TRANSPORT_AND_EFFLUX	33	0.43678012	1.4816378	0.04477612	1	0.999	4339	tags=42%, list=24%, signal=56%
BROWNE_HCMV_INFECTION_6HR_DN	157	0.3272081	1.4804101	0.04731861	1	0.999	4396	tags=42%, list=24%, signal=55%
LEE_AGING_NEOCORTEX_DN	51	0.34871122	1.4739994	0.022653721	1	0.999	2178	tags=27%, list=12%, signal=31%
MARTORIATI_MDM4_TARGETS_FETAL_LIVER_DN	479	0.31409332	1.4693968	0.043062203	1	0.999	4417	tags=37%, list=25%, signal=47%
HOWLIN_CITED1_TARGETS_1_UP	33	0.3844786	1.459304	0.04373178	1	0.999	2861	tags=27%, list=16%, signal=32%
SHIPP_DLBCL_CURED_VS_FATAL_UP	29	0.38754874	1.448318	0.04661017	1	0.999	2415	tags=31%, list=13%, signal=36%
KONDO_EZH2_TARGETS	192	0.26440793	1.3345587	0.04477612	1	1	3033	tags=26%, list=17%, signal=30%

Supplementary Table 3: Downregulated pathways in *RUNX1*-mutated AML

Pathway	Size	ES	NES	NOM p-val	FDR q-val	FWER p-val	Rank at max	Leading edge
BIOCARTA_MITOCHONDRIA_PATHWAY	19	-0.7161914	-2.039181	0	0.25681373	0.173	1719	tags=53%, list=10%, signal=58%
REACTOME_GLYCOSPHINGOLIPID_METABOLISM	39	-0.60526234	-1.8162571	0.0033955858	1	0.872	743	tags=31%, list=4%, signal=32%
WP_NANOMATERIAL_INDUCED_APOPTOSIS	20	-0.6128473	-1.7895322	0.0055555557	1	0.916	2946	tags=45%, list=16%, signal=54%
WAGNER_APO2_SENSITIVITY	18	-0.6235822	-1.7509547	0.008695652	1	0.965	1263	tags=33%, list=7%, signal=36%
WP_APOPTOSIS_MODULATION_BY_HSP70	19	-0.6269025	-1.7266896	0.013257576	1	0.981	2946	tags=47%, list=16%, signal=57%
KEGG_GLYCOSAMINOGLYCAN_DEGRADATION	18	-0.6867038	-1.6856623	0.010638298	1	0.994	2984	tags=56%, list=17%, signal=67%
WP_GLYCOSAMINOGLYCAN_DEGRADATION	15	-0.69961977	-1.6617068	0.03068592	1	0.997	2984	tags=60%, list=17%, signal=72%
ROYLANCE_BREAST_CANCER_16Q_COPY_NUMBER_DN	19	-0.5919067	-1.6546073	0.029513888	1	0.997	1730	tags=53%, list=10%, signal=58%
WP_METHIONINE_DE_NOVO_AND_SALVAGE_PATHWAY	19	-0.597519	-1.6521629	0.021032505	1	0.997	3757	tags=58%, list=21%, signal=73%
KEGG_OTHER_GLYCAN_DEGRADATION	15	-0.6779646	-1.6257278	0.04708098	1	0.999	1282	tags=53%, list=7%, signal=57%
WANG_CLASSIC_ADIPOGENIC_TARGETS_OF_PPARG	24	-0.5626743	-1.5848125	0.016313214	1	1	5374	tags=71%, list=30%, signal=101%
REACTOME_HYALURONAN_METABOLISM	16	-0.6030546	-1.5688671	0.049099836	1	1	323	tags=31%, list=2%, signal=32%
REACTOME_BBSOME_MEDIATED_CARGO_TARGETING_TO_CILIUM	23	-0.5820366	-1.5460275	0.04518664	1	1	5741	tags=65%, list=32%, signal=96%
REACTOME_BLOOD_GROUP_SYSTEMS_BIOSYNTHESIS	17	-0.5470982	-1.5379107	0.024853801	1	1	1263	tags=29%, list=7%, signal=32%
ZHAN_MULTIPLE_MYELOMA_CD1_AND_CD2_DN	54	-0.3959537	-1.5377092	0.02247191	1	1	3365	tags=39%, list=19%, signal=48%
MOREIRA_RESPONSE_TO_TSA_UP	28	-0.47837305	-1.5352522	0.039049234	1	1	5186	tags=54%, list=29%, signal=75%
BILANGES_SERUM_SENSITIVE_VIA_TSC1	15	-0.5335756	-1.5202199	0.036363635	1	1	2823	tags=53%, list=16%, signal=63%
KEGG_AMYOTROPHIC_LATERAL_SCLEROSIS_ALS	41	-0.44808972	-1.5107236	0.036585364	1	1	3095	tags=37%, list=17%, signal=44%
BOYAULT_LIVER_CANCER_SUBCLASS_G123_DN	42	-0.440007	-1.5103427	0.030165913	1	1	3353	tags=38%, list=19%, signal=47%
REACTOME_KERATAN_SULFATE_KERATIN_METABOLISM	27	-0.47758374	-1.5037926	0.028753994	1	1	2984	tags=41%, list=17%, signal=49%
RUAN_RESPONSE_TO_TROGLITAZONE_UP	20	-0.49625146	-1.4982431	0.036624204	1	1	2270	tags=40%, list=13%, signal=46%
YEGNASUBRAMANIAN_PROSTATE_CANCER	93	-0.40100428	-1.4921252	0.04858934	1	1	3293	tags=32%, list=18%, signal=39%
WP_SARSCOV2_MITOCHONDRIAL_CHRONIC_OXIDATIVE_STRESS_AND_ENDOTHELIAL_DYSFUNCTION	25	-0.46568382	-1.4850926	0.04553734	1	1	5151	tags=64%, list=29%, signal=90%
REACTOME_PHASE_I_FUNCTIONALIZATION_OF_COMPOUNDS	65	-0.4158694	-1.4746938	0.030259365	1	1	3674	tags=40%, list=20%, signal=50%
WP_PARKINSONS_DISEASE_PATHWAY	35	-0.42404723	-1.4475983	0.042955328	1	1	2935	tags=37%, list=16%, signal=44%
ROSS_AML_WITH_PML_RARA_FUSION	79	-0.58043605	-1.4379978	0.044262294	1	1	3229	tags=48%, list=18%, signal=58%
REACTOME_BIOLOGICAL_OXIDATIONS	139	-0.3832166	-1.4340707	0.0474732	1	1	3757	tags=37%, list=21%, signal=47%

Supplementary Table 4: FCM result in patients' with *PHF6* mutation

Cohort ID	Age Group	Sex	Dx	Subtype	Karyotype	cDNA	AA	VAF	TdT	GCSAM	LY9
PHF6_0057	70-80	M	AML	sAML	Trisomy 8	c.953C>G	p.S318X	0.89	Neg	Pos (dim)	Pos
PHF6_0060	50-60	M	AML	pAML	Other	c.862G>A	p.A288T	1	Pos	Neg	Neg
PHF6_0370	70-80	M	AML	sAML	Trisomy 8	c.908A>G	p.Y303C	0.8333	Pos	Neg	Neg
PHF6_0692	60-70	M	AML	sAML	Normal	c.385C>T	p.R129X	0.9256	Neg	Pos (dim)	Neg
PHF6_0992	50-60	F	AML	sAML	DelX	c.820C>T	p.R274X	1	Neg	Neg	Pos
PHF6_1085	60-70	M	AML	pAML	Normal	c.712_714delGCCinsTGA	p.A238X	0.933	Neg	Neg	Neg

Supplementary Table 5 The number of cases by each cohort

Cohort	Diseases	Number of cases
CCF	All	1465
	AML	1039
	MDS	208
	MDS/MPN	42
	MPN	176
MLL	All	5109
	AML	4007
	MDS	706
	MDS/MPN	335
	MPN	61
BeatAML	All	618
	AML	589
	MDS	17
	MDS/MPN	9
	MPN	3
German–Austrian AML Study	AML	1251

Supplementary Table 6 Targeted panel genes

Gene	Transcript	Exon
ABL1	NM_005157.5	4-6
ASXL1	NM_15338.5	10-13
BCOR	NM_17745.5	2-15
BCORL1	NM_021946.4	1-12
BRAF	NM_004333.4	15
CALR	NM_004343.3	9
CBL	NM_005188.3	8-9
CDKN2A	NM_000077.4	1-2
CDKN2A	NM_058195.3	1
CEBPA	NM_004364.4	1
CSF3R	NM_000760.3	14-17
CUX1	NM_001202543.1	15-24
CUX1	NM_001913.4	1-23
DDX41	NM_016222.3	1-17
DNMT3A	NM_022552.4	2-23
EED	NM_003797.4	1-12
ETNK1	NM_018638.4	3
ETV6	NM_001987.4	1-8
EZH2	NM_004456.4	2-20
FBXW7	NM_018315.4	7-11
FLT3	NM_004119.2	14-17, 19-20
GATA1	NM_002049.3	2, 4
GATA2	NM_032638.4	2-6
GNAS	NM_000516.5	8-11
IDH1	NM_005896.3	4
IDH2	NM_002168.3	4
IKZF1	NM_006060.5	2-3, 5-7
JAK2	NM_004972.3	12-16
JAK3	NM_000215.3	11-18
KDM6A	NM_021140.3	1-29
KIT	NM_000222.2	2, 8-11, 13, 17
KMT2A	NM_005933.3	1-36
KRAS	NM_004985.4	2-4
LUC7L2	NM_001244585.1	2-11
MPL	NM_005373.2	10-11
MYD88	NM_002468.4	5
NF1	NM_000267.3	1-57
NF1	NM_001042492.2	31
NOTCH1	NM_17617.4	26, 27, 34
NPM1	NM_002520.6	8-11
NRAS	NM_002524.4	2-4
PAX5	NM_016734.2	1-10
PHF6	NM_001015877.1	2-10
PIGA	NM_002641.3	2-6
PPM1D	NM_003620.3	1-6
PRPF8	NM_006445.3	2-43
PTEN	NM_000314.6	1-9
PTPN11	NM_002834.3	3-4, 12-13
RAD21	NM_006265.2	2-14
RIT1	NM_006912.5	5
RUNX1	NM_001754.4	2-9
RUNX1	NM_001122607.1	5
SETBP1	NM_015559.2	4
SF3B1	NM_012433.3	13-16
SH2B3	NM_005475.2	2
SMC1A	NM_006306.3	1-25
SMC3	NM_005445.3	1-29
SRSF2	NM_003016.4	1-2
STAG2	NM_00104279.2	3-35
STAT3	NM_003150.3	20-21
STAT5B	NM_012448.3	16-18
SUZ12	NM_015355.3	1-16
TET2	NM_001127208.2	3-11
TP53	NM_000546.5	2-11
U2AF1	NM_006758.2	2, 6
WT1	NM_000378.4	1-9
ZRSR2	NM_005089.3	1-11
