# Supplementary material for: Molecular and clinical analyses of PHF6 mutant myeloid neoplasia provide their pathogenesis and therapeutic targeting
Source: Nat Commun. 2024 Feb 28;15:1832. doi: 10.1038/s41467-024-46134-w (PMC10901781; doi:10.1038/s41467-024-46134-w)
Supplement: Supplementary file 3 — Description of Additional Supplementary Files [file 41467_2024_46134_MOESM3_ESM.docx]

**Description of Additional Supplementary Files**

Supplementary Data 1

Description: Patients' characteristics in this study

Supplementary Data 2

Description: Detected mutations in all patients

Supplementary Data 3

Description: Co-immunoprecipitated proteins with PHF6

Supplementary Data 4

Description: Peaks in ChIPseq with PHF6 antibody

Supplementary Data 5

Description: Peaks in ChIPseq with RUNX1 antibody

Supplementary Data 6

Description: Peaks in ChIPseq with control IgG

Supplementary Data 7

Description: Differential expressed genes in *PHF6*-mutated AML

Supplementary Data 8

Description: Downregulated pathways in *PHF6*-mutated AML

Supplementary Data 9

Description: Differential expressed genes in *RUNX1*-mutated AML

Supplementary Data 10

Description: Upregulated pathways in *RUNX1*-mutated AML
