# Supplementary material for: Molecular and clinical analyses of PHF6 mutant myeloid neoplasia provide their pathogenesis and therapeutic targeting
Source: Nat Commun. 2024 Feb 28;15:1832. doi: 10.1038/s41467-024-46134-w (PMC10901781; doi:10.1038/s41467-024-46134-w)
Supplement: Supplementary file 14 — Reporting Summary [file 41467_2024_46134_MOESM14_ESM.pdf]

nature portfolio

Corresponding author(s): Jaroslaw P. Maciejewski

Last updated by author(s): Dec 21, 2023

Reporting Summary

Nature Portfolio wishes to improve the reproducibility of the work that we publish. This form provides structure for consistency and transparency in reporting. For further information on Nature Portfolio policies, see our Editorial Policies and the Editorial Policy Checklist.

Statistics

For all statistical analyses, confirm that the following items are present in the figure legend, table legend, main text, or Methods section.

n/a	Confirmed	
☐	☒	The exact sample size (*n*) for each experimental group/condition, given as a discrete number and unit of measurement
☐	☒	A statement on whether measurements were taken from distinct samples or whether the same sample was measured repeatedly
☐	☒	The statistical test(s) used AND whether they are one- or two-sided<br>*Only common tests should be described solely by name; describe more complex techniques in the Methods section.*
☐	☒	A description of all covariates tested
☐	☒	A description of any assumptions or corrections, such as tests of normality and adjustment for multiple comparisons
☐	☒	A full description of the statistical parameters including central tendency (e.g. means) or other basic estimates (e.g. regression coefficient) AND variation (e.g. standard deviation) or associated estimates of uncertainty (e.g. confidence intervals)
☐	☒	For null hypothesis testing, the test statistic (e.g. *F*, *t*, *r*) with confidence intervals, effect sizes, degrees of freedom and *P* value noted<br>*Give P values as exact values whenever suitable.*
☒	☐	For Bayesian analysis, information on the choice of priors and Markov chain Monte Carlo settings
☒	☐	For hierarchical and complex designs, identification of the appropriate level for tests and full reporting of outcomes
☒	☐	Estimates of effect sizes (e.g. Cohen's *d*, Pearson's *r*), indicating how they were calculated

Our web collection on statistics for biologists contains articles on many of the points above.

Software and code

Policy information about availability of computer code

Data collection	No software was used for data collection.
Data analysis	Scripts for data processing and analysis of RNA-sequencing are available at http://github.com/ysokbt/PHF6

For manuscripts utilizing custom algorithms or software that are central to the research but not yet described in published literature, software must be made available to editors and reviewers. We strongly encourage code deposition in a community repository (e.g. GitHub). See the Nature Portfolio guidelines for submitting code & software for further information.

Data

Policy information about availability of data

All manuscripts must include a data availability statement. This statement should provide the following information, where applicable:

- Accession codes, unique identifiers, or web links for publicly available datasets
- A description of any restrictions on data availability
- For clinical datasets or third party data, please ensure that the statement adheres to our policy

All the data supporting these findings, including DNA sequencing, ChIPseq, and the mass spec data were deposited in the dbGaP (accession number, phs001898.v1.p1 and phs003303.v1.p1), GEO (accession number, GSE229948), and the ProteomeXchange consortium (accession number, PXD042441), respectively. Molecular annotation is provided in the supplemental tables of the submitted manuscript. Supplementary Tables 1, 3, and 4 contain a list of patients with detailed annotation of karyotype, mutational status, and proteomics data, respectively. Public series (the BEAT AML Master Trial and the German-Austrian cohorts) are available in the respective published articles. Part of the data were extracted from open-access sources: https://github.com/ardadurmaz/mds_latent;

https://github.com/ardadurmaz/aml). Whole genome sequencing data can be requested from the Munich Leukemia laboratory (torsten.haferlach@mll.com). All relevant data are available from the authors upon request by contacted the corresponding author: maciejj@ccf.org.

Research involving human participants, their data, or biological material

Policy information about studies with human participants or human data. See also policy information about sex, gender (identity/presentation), and sexual orientation and race, ethnicity and racism.

Reporting on sex and gender	Sex was determined based on self-reporting and we checked for a match to the chromosomal test results.
Reporting on race, ethnicity, or other socially relevant groupings	We enrolled 1465 and 5109 myeloid neoplasm patients at the Cleveland Clinic and Munich Leukemia Laboratory, respectively. The characteristics of the patients are summarized in Supplementary Table 1.
Population characteristics	A cohort of 8,443 myeloid neoplasm (MN) patients (148 MNs with PHF6 mutations and 8,295 MNs without PHF6 mutations) was used as study cohort. Considering the rarity of PHF6 mutations in myeloid neoplasms, no criteria were established based on disease type, age, or sex.
Recruitment	Considering the rarity of PHF6 mutations in myeloid neoplasms, a total of 8,443 consecutive patients with MNs were selected for the purpose of the study. To avoid self-selection bias, no criteria were established based on disease type, age, or sex.
Ethics oversight	This research was conducted under the Institutional Review Boards of Cleveland Clinic Foundation (IRB #5024). Specimens were collected after receiving written informed consent in accordance with the Declaration of Helsinki and in agreement with IRBs of the participating institutions.

Note that full information on the approval of the study protocol must also be provided in the manuscript.

Field-specific reporting

Please select the one below that is the best fit for your research. If you are not sure, read the appropriate sections before making your selection.

- [x] Life sciences
- [ ] Behavioural & social sciences
- [ ] Ecological, evolutionary & environmental sciences

For a reference copy of the document with all sections, see nature.com/documents/nr-reporting-summary-flat.pdf

Life sciences study design

All studies must disclose on these points even when the disclosure is negative.

Sample size	No prior sample size calculations were performed. Considering the rarity of PHF6 mutations in myeloid neoplasms, a total of 8,443 consecutive patients with myeloid neoplasms were selected for the purpose of the study.
Data exclusions	All patients were suitable for analyses and no data were excluded.
Replication	The genomic data of our cohort were consistent with previous findings in similar populations. In addition to avoid variability and bias, same specimens (bone marrow cells) were used once as source of genomic information.
Randomization	Patient randomization is not relevant for this study. All patient samples were collected at diagnosis and underwent no prior treatment/ randomization.
Blinding	Investigators were not blind to mutated groups as knowledge of mutations in cases and cell lines was necessary to perform the experiments and analyses.

Reporting for specific materials, systems and methods

We require information from authors about some types of materials, experimental systems and methods used in many studies. Here, indicate whether each material, system or method listed is relevant to your study. If you are not sure if a list item applies to your research, read the appropriate section before selecting a response.

Materials & experimental systems

n/a	Involved in the study	
	x	Antibodies
	x	Eukaryotic cell lines
x		Palaeontology and archaeology
x		Animals and other organisms
	x	Clinical data
x		Dual use research of concern
x		Plants

Methods

n/a	Involved in the study	
	x	ChIP-seq
	x	Flow cytometry
x		MRI-based neuroimaging

Antibodies

Antibodies used

Antibodies are described in the Methods of the manuscript and in addition they are listed below:

For ChIPseq:
PHF6 (SCBT, sc-365237AC)

For Immunohistochemistry
PHF6 (Millipore Sigma, HPA001023, 1:25)
LY9 (Abcam, EBR22611-91, 1:50)
GCSAM (Abcam, EPR14333, 1:500)

Immunoprecipitation, Western blot
PHF6 (SCBT, sc-365237AC)

Validation

All antibodies are commercially available and were validated according to manufactures' instructions.
PHF6 (SCBT, sc-365237AC), Reactive Species: Human, Mouse, Rat, Application: IP, IHC, WB
PHF6 (Millipore Sigma, HPA001023), Reactive Species: Human, Application IHC
LY9 (Abcam, EBR22611-91), Reactive Species: Mouse, Rat, Human, Application: WB, IHC
GCSAM (Abcam, EPR14333), Reactive Species: Human, Application, IHC, FACS

Eukaryotic cell lines

Policy information about cell lines and Sex and Gender in Research

Cell line source(s)	THP-1 cells were purchased from ATCC (Cat# TIB-202).
Authentication	STR profile analysis.
Mycoplasma contamination	Routinely tested negative for mycoplasma contamination.
Commonly misidentified lines (See ICLAC register)	We do not use commonly misidentified cell lines.

Clinical data

Policy information about clinical studies

All manuscripts should comply with the ICMJEguidelines for publication of clinical research and a completedCONSORT checklist must be included with all submissions.

Clinical trial registration	N.A.
Study protocol	N.A.
Data collection	Samples were collected between 09/2005 and 08/2022.
Outcomes	The overall survival was defined from diagnosis to death or last follow-up.

Plants

Seed stocks	N.A.
Novel plant genotypes	N.A.
Authentication	N.A.

ChIP-seq

Data deposition

- [x] Confirm that both raw and final processed data have been deposited in a public database such as GEO.
- [x] Confirm that you have deposited or provided access to graph files (e.g. BED files) for the called peaks.

Data access links<br>*May remain private before publication.*	GEO (accession number, GSE229948)
Files in database submission	THP1_PHF6.bam<br>THP1_PHF6.bai<br>THP1_PHF6.bw<br>THP1_PHF6.bed
Genome browser session<br>(e.g. UCSC)	N.A.

Methodology

Replicates	ChIPseq was performed in duplicate with independent cell cultures. IP and sequencing library preparation were performed at different points in time.
Sequencing depth	Libraries of the precipitated DNA were sequenced on Illumina HiSeq 2500 using 50 bp single-end mode.
Antibodies	PHF6 (SCBT, sc-365237AC)
Peak calling parameters	Sequencing data were mapped to hg19 using Bowtie2 with the default parameters. Each peak were called using HOMER with the default parameters.
Data quality	For co-localization analysis of PHF6 and RUNX1, we used peaks with FDR <0.05.
Software	Bowtie2 (2.5.1); HOMER (4.11); deeptools (3.5.1)

Flow Cytometry

Plots

Confirm that:

- [x] The axis labels state the marker and fluorochrome used (e.g. CD4-FITC).
- [x] The axis scales are clearly visible. Include numbers along axes only for bottom left plot of group (a 'group' is an analysis of identical markers).
- [x] All plots are contour plots with outliers or pseudocolor plots.
- [x] A numerical value for number of cells or percentage (with statistics) is provided.

Methodology

Sample preparation	Bone marrow cells were used for AML immunophenotyping. Flow cytometry was conducted at the Pathology and Laboratory Medicine Institute at the Cleveland Clinic.
Instrument	BD LSRII and Fortessa
Software	FlowJo™ v10 Software
Cell population abundance	Cell sorting was not performed. Experiments were conducted to define the percentage of positive cells per each marker analyzed.
Gating strategy	AML cell markers analyzed were: CD7/CD5; CD8/CD4; CD7/CD3; CD10/CD19; CD20/CD10; CD19/CD22; CD19/CD34; CD13/CD34; CD33/CD34; CD65/CD13; HLADR/CD34; CD16/CD13; CD64/CD14; CD16/CD11b; CD56/CD2; CD117/CD38; CD14/CD56; CD45/7AAD. Proportion of cells was used for AML immunophenotyping. The flow plot for CD13 versus TdT is shown in Figure 4.

- [ ] Tick this box to confirm that a figure exemplifying the gating strategy is provided in the Supplementary Information.
